# Predictability of thermal fluctuations influences functional traits of a cosmopolitan marine diatom

**DOI:** 10.1098/rspb.2021.2581

**Published:** 2022-04-27

**Authors:** Raissa L. Gill, Sinead Collins, Phoebe A. Argyle, Michaela E. Larsson, Robert Fleck, Martina A. Doblin

**Affiliations:** ^1^ Climate Change Cluster, Faculty of Science, University of Technology Sydney, NSW, Australia; ^2^ School of Life Sciences, Faculty of Science, University of Technology Sydney, NSW, Australia; ^3^ Institute for Evolutionary Biology, University of Edinburgh, EH8 9YL, UK; ^4^ Sydney Institute of Marine Science, Mosman, NSW, Australia

**Keywords:** phytoplankton, diatoms, phenotypic plasticity, ocean warming, climate impact, primary production

## Abstract

Evolutionary theory predicts that organismal plasticity should evolve in environments that fluctuate regularly. However, in environments that fluctuate less predictably, plasticity may be constrained because environmental cues become less reliable for expressing the optimum phenotype. Here, we examine how the predictability of +5°C temperature fluctuations impacts the phenotype of the marine diatom *Thalassiosira pseudonana*. Thermal regimes were informed by temperatures experienced by microbes in an ocean simulation and featured regular or irregular temporal sequences of fluctuations that induced mild physiological stress. Physiological traits (growth, cell size, complexity and pigmentation) were quantified at the individual cell level using flow cytometry. Changes in cellular complexity emerged as the first impact of predictability after only 8–11 days, followed by deleterious impacts on growth on days 13–16. Specifically, cells with a history of irregular fluctuation exposure exhibited a 50% reduction in growth compared with the stable reference environment, while growth was 3–18 times higher when fluctuations were regular. We observed no evidence of heat hardening (increasingly positive growth) with recurrent fluctuations. This study demonstrates that unpredictable temperature fluctuations impact this cosmopolitan diatom under ecologically relevant time frames, suggesting shifts in environmental stochasticity under a changing climate could have widespread consequences among ocean primary producers.

## 1. Introduction

Climate change is characterized by an array of alterations in Earth's physical and chemical properties and is shifting the way organisms experience the natural world. Notably, atmospheric warming is associated with an increase in the frequency, intensity and/or variability of extreme weather events, such as heatwaves, heavy precipitation, cyclones, droughts, wildfires and floods [1]. An organism's response to environmental variation depends on the shape of its reaction norm—the relationship between phenotypic trait change and environmental change. Reaction norms are commonly used to understand and model both species distributions and organismal responses to environmental change. Importantly, the pattern and extent of environmental variability that populations experience over time influences the shape of reaction norms [2]. While there is a developed body of theory about how past environmental experience affects plasticity [3], empirical studies of key organisms and relevant environmental changes [4–6] are crucial for validating the theory and predicting how climate change will shape the future biosphere.

Environmental variation can be characterized by the amplitude and frequency of changes in environmental properties [7], but a key aspect driving plasticity and evolution of populations is environmental predictability [8]. Colwell *et al*. [9] defined this as the regularity of a phenomenon that occurs periodically through time [9], where the reliability of an environmental cue can be instrumental to expressing the optimum phenotype or behaviour in response to a current and/or future driver [10]. Highly reliable environmental cues are characterized by temporally predictable sequences of environmental change, such as regularly occurring seasonal temperature variations which signal migration in birds [11]. Contrastingly, cues that occur as unpredictable sequences can impair an organism's ability to assess or forecast changes in their environment, where irregular cues can lead to a mismatch with the organisms phenotype and less effective or even maladaptive behaviours [12]. As environmental variables are expected to become increasingly unpredictable with ongoing greenhouse gas emissions [1], empirical support for and model validation of the relationship between predictability of environmental change and organismal responses [2,8,13] are vital. Studies to date have shown that less predictable environments lead to lower plasticity in organisms such as *Dunaliella salina* (a unicellular, halotolerant microalga), where increasingly unpredictable fluctuations in salinity caused decreased growth, more extinctions and/or reduced morphological plasticity [14,15]. However, studies of this nature are relatively scarce compared with those altering the amplitude and/or frequency of environmental changes. Thus, environmental predictability was selected as the component of variation for manipulation in this study.

The physiology underlying organismal responses to environmental variability can vary considerably depending on the nature of the fluctuations [16]. For instance, if the amplitude exceeds the acute or cumulative stress threshold of an organism, a cellular stress response is initiated, which may involve increased production of reactive oxygen species (ROS), diversion of energy towards cellular repair and/or the upregulation of stress response genes [17,18]. Any subsequent exposure requires additional energy allocation to repair cellular damage and to sustain tolerance [19], which can become lethal if exposure occurs at high frequencies with inadequate intervals for recovery between events [20]. Repeated exposure to a stressor can have bidirectional effects on an organism's survivability: either it contributes additively to stress and has a negative impact on subsequent recovery, or it may prime the organism to be more resilient in the face of subsequent stressors, referred to as cross-protection, cross-tolerance, stress imprint, stress memory or hardening [21–25]. Additionally, these effects can occur concurrently with stress accumulation—for instance, Samuels *et al*. [26] demonstrated both heat hardening and accumulated stress in *Actinocyclus actinochilus* populations (Southern Ocean diatoms) acclimated to sub-lethal temperatures prior to heatwave exposure [26]. In fast-growing unicellular organisms such as marine microbes, reproduction by binary fission and subsequent transgenerational plasticity may also provide a mechanism whereby a population is hardened to warming through previous ancestral exposures [27], analogous to the hardening of individual organisms such as plants with longer generation times.

Marine phytoplankton are a diverse group of microbes that inhabit the upper ocean, where they harness light energy for photosynthesis that is then distributed to the ocean food web as organic carbon [28]. These organisms experience enhanced variation in environmental conditions compared to benthic organisms because they drift in ocean currents [29]. Exposure to environmental variation, short generation times and amenability to experimentation make marine phytoplankton excellent candidates for investigating the implications of environmental variability.

Here, we examine the effect of environmental predictability and history on growth and other functional traits in the cosmopolitan marine diatom *Thalassiosira pseudonana* using fluctuating temperature regimes. Temperature was selected as the fluctuating environmental value for this study as it varies on multiple temporal scales in the ocean and influences the global distribution of marine phytoplankton [30,31]. We hypothesized that irregular sequences of temperature change would negatively impact *T. pseudonana* growth with associated changes in the phenotype, largely due to limited opportunity to anticipate warming under temporally variable timescales. We also examined whether there was evidence of hardening as *T. pseudonana* experienced cumulative fluctuations.

## 2. Methods

### (a) Experimental design

To design experimental thermal regimes that represented environmentally relevant patterns of predictability in temperature fluctuations from the perspective of planktonic marine microbes, we used data collected as part of the oceanographic citizen science project ‘Adrift’ (adrift-project.com). Adrift uses a Lagrangian ocean analysis framework [32] to track passive particle movement through the uppermost surface layer of a dynamic three-dimensional numerical ocean model. An upwards fluctuation of +5°C was selected from a baseline of 18°C, informed by the greatest seasonal variation in temperature experienced by virtual microbes drifting in the core flow of the Eastern Australian Current in austral Autumn of 2017. The 18°C baseline represents the lowest temperature experienced during austral Autumn and is well below the estimated thermal optimum (*T*_opt_) for *T. pseudonana*. Boyd *et al*. [33] observed *T*_opt_ of 25°C with instances of mortality at greater than or equal to 30°C among six strains of *T. pseudonana* tested across temperatures spanning 10 to 32.5°C [33]. For nutrients, the maximum concentration of nitrate experienced among virtual microbes in Autumn (3.74 µmol l^−1^) was selected for daily dosing in experiments, representing the best-case scenario for the availability of this limiting nutrient in this ocean region. More details on the Adrift model, simulation procedure and data processing are provided in the supporting information (see electronic supplementary material, adrift methods section and figure S1).

Thermal regimes had two phases. In the first phase (test of environmental predictability), four upwards temperature fluctuations were simulated with the same magnitude, frequency and period of thermal exposure, but differing in the sequence of intervals between them (1 and/or 2 days). Interval sequences were spaced regularly or irregularly through time to simulate temporally predictable and unpredictable environmental conditions, respectively. In the second phase (test of environmental history), there was a fifth and final fluctuation that was out of sequence with the preceding fluctuations, occurring after a 4-day interval. The purpose of this second phase was to test how the predictability of temperature fluctuations in *T. pseudonana*'s recent history impacts its response to an additional, unanticipated fluctuation. A control regime maintained at 18°C was included to allow relative comparisons to stable temperature as well as the standardization of responses among regimes. The thermal regimes are listed below (see also electronic supplementary material, figure S2) where for regime labels, ‘R’ denotes regular fluctuations in temperature, ‘I’ denotes irregular fluctuations, and the proceeding numbers represent the intervals (in days) between each fluctuation:

(1) Control; no fluctuations, maintained at base temperature of 18°C.
(2) R-1114; regular fluctuations of +5°C (18 to 23°C) with equal 1-day intervals between fluctuations 1–4, followed by a 4-day interval preceding fluctuation 5
(3) R-2224; regular fluctuations with equal 2-day intervals between fluctuations 1–4, followed by a 4-day interval preceding fluctuation 5
(4) I-1124; irregular fluctuations with mixed 1- and 2-day intervals between fluctuations 1–4 (2-day interval last in sequence), followed by a 4-day interval preceding fluctuation 5
(5) I-2114; irregular fluctuations with mixed 1- and 2-day intervals between fluctuations 1–4 (2-day interval first in sequence), followed by a 4-day interval preceding fluctuation 5

To isolate the effect of environmental predictability from recovery capacity, the regimes were designed so that R-1114 and R-2224 featured the lowest and highest cumulative windows of time at 18°C between successive fluctuations. Thus, if recovery capacity was the major driver negatively impacting *T. pseudonana* responses, R-1114 would be impacted the greatest (total of 7 days at 18°C between fluctuations 1–5), followed by I-1124 and I-2114 (8 days) and finally R-2224 (10 days). However, if the irregular regimes were most impacted, it would provide evidence for environmental predictability being the driving factor. Our observation was that I-2114 and I-1124 were most negatively impacted, indicating the major driver was predictability, not recovery.

### (b) *Thalassiosira pseudonana* culturing and experimental set-up

A strain of *T. pseudonana* (CCMP 3367) was obtained in August 2018 from the Provasoli-Guillard National Center of Marine Phytoplankton (NCMA, formerly known as the CCMP; https://ncma.bigelow.org/) and grown in artificial seawater (ASW, salinity 35 ppt) [34] with f/2 nutrients [35]. Stock culture was maintained in exponential growth at 23–26°C (monitored using iButton devices (DS1921G), Thermochron, Australia) under a 12 : 12 light/dark cycle, with approximately 60 µmol photons m^−2^ s^−1^ cool white fluorescent light (monitored using a light meter (LI-250A) and quantum sensor (LI-190R), LI-COR Biosciences, NE, USA) in 500 ml wide-mouth Erlenmeyer flasks. Flasks were agitated daily to prevent settling.

One hour prior to initiating experiments, an aliquot of the *T. pseudonana* stock culture was centrifuged (1475 RCF, 5 min) to separate cells from the medium, and cells were resuspended in fresh ASW with f/2 nutrients excluding nitrate (NaNO_3_) (henceforth f/2-N medium) by gentle agitation. Pilot studies demonstrated no visual deterioration in cell number or quality for up to 24 h post-centrifugation. Cells were then inoculated into sterile 12-multi-well plates (Falcon, Corning, NY, USA) to a final concentration of 5000 cells ml^−1^ in f/2-N medium. Plates were covered with a sealing membrane (Breathe-Easy, Diversified Biotech, MA, USA) to minimize evaporation. A small slit was made in the membrane covering each well to allow daily NaNO_3_ dosing to a final concentration of 3.74 µmol l^−1^ (2.2 µl of 3740 µmol l^−1^ NaNO_3_ stock solution). A pilot study demonstrated there were no between-plate effects under a similar experimental set-up comparing four plates with wells treated as individual replicates (ANOVA: *F*_3,44_ = 1.50, *p* = 0.23; *n* = 12, data not shown). Therefore, each of the regimes was allocated to a single plate and wells were treated independently, yielding 12 replicates per regime.

### (c) Experimental execution and sampling

Following inoculation, populations were exposed to 18°C for 60 h before the onset of fluctuations. Regimes were simulated by moving plates manually between two identical growth incubators (520 L Climatron, Thermoline Scientific, Australia) at 10.00 and 20.00 (10 h shift) during the light period, with controls also being relocated within the same incubator. Plates were distributed randomly across their appropriate incubator shelf at each sampling point to incorporate any light variation effects. This was achieved by assigning them using a random number generator to one of six quadrats drawn across the incubator shelves. Incubators had *in-situ* temperatures of 17.85 ± 1.60°C and 23.62 ± 1.76°C (mean ± s.d.), respectively, and were illuminated with 200 ± 20 µmol photons m^−2^ s^−1^ cool white fluorescent light under a 12 : 12 square-wave light/dark cycle (lights on at 09.00, off at 21.00). Plates were incubated under static water conditions (i.e. no shaking).

At 10.00 ± 00.15 and 20.00 ± 00.15 (preceding and succeeding thermal fluctuations), wells were gently mixed and subsampled by aliquoting 20 µl of culture into a 96-multi-well plate (Falcon, Corning, NY, USA), and cells were preserved with paraformaldehyde (PFA, 1% v/v final concentration) (Electron Microscopy Sciences, Ft. Washington, PA, USA). Samples taken at 10.00 were stored in a 4°C refrigerator for up to 12 h. Cell abundance and traits were quantified with a flow cytometer (CytoFLEX LX, Beckman Coulter, CA, USA) each evening.

To ensure cells did not reach stationary phase prematurely, wells were diluted each evening at 22.30, where the removed sample volume (total of 40 µl per well per day) was replaced with fresh f/2-N medium (approx. 18% daily dilution). If the mean cell abundance of the plate exceeded 5 × 10^4^ cells ml^−1^ at the evening's flow cytometric cell count, a 50% dilution was applied instead. This process ensured populations remained in exponential growth phase throughout the entire experiment. Wells received their daily dose of NaNO_3_ at 23.30 following dilutions. Replacement media was pre-warmed to approximately 20°C prior to dilutions.

### (d) Flow cytometry-based trait determination

*Thalassiosira pseudonana* populations were enumerated flow cytometrically (minimum cell count of 100) and gated by their red fluorescence and side scatter (SSC) signals with a threshold of 4000 on forward scatter (FSC). The photosynthetic pigment chlorophyll-*a* (Chl-A) was estimated per cell using the fluorescence emission (690/50 nm detection) induced by blue excitation (488 nm), normalized to the median fluorescence of standard yellow-green fluorescent beads (1 µm FluroSpheres, Life Technologies, CA, USA) which were added to cell-free culture medium and analysed immediately before samples. Cell size and complexity (i.e. external granularity and/or internal vacuoles) was inferred from FSC and SSC, respectively, normalized to the median values of calibration beads (1 µm Flow Cytometry Size Calibration Kit, Thermofisher, MA, USA). Standard quality control beads (CYTOFLEX LX Daily QC Fluorospheres, Beckman Coulter, CA, USA) were run prior to each session to ensure measurements across the experimental time frame were comparable.

### (e) Reactive oxygen species production

To determine whether the increase in temperature from 18 to 23°C induced a physiological stress response, we assessed *T. pseudonana*'s production of ROS, an indicator of oxidative damage and photosynthetic inhibition [36–38]. Details on this experiment and the protocol used are provided in the electronic supplementary material, ROS production methods section. Normalized ROS-induced fluorescence per cell was greater at 20.5 and 23°C relative to 18°C (ANOVA: *F*_2,33_ = 19.23, *p* < 0.0001 with Tukey HSD: *p*_adj_ < 0.0001 in both cases), indicating that the fluctuations caused mild physiological stress (electronic supplementary material, figure S3).

### (f) Statistical and visual analysis of data

To examine how *T. pseudonana* responded to each temperature fluctuation within regimes, growth rate (cell divisions per hour) and proportional changes in median cell size (estimated by FSC), complexity (SSC) and chlorophyll-*a* (Chl-A) per cell were calculated from trait values preceding and succeeding each fluctuation. Growth rates were calculated using equation (2.1) below, where *N*_0_ and *N* denote the cell abundance at 10.00 and 20.00, respectively, and *t*_0_ and *t* denote time in hours (*t*_0_ = 0 and *t* = 10 h, respectively). Functional traits were calculated using equation (2.2) below, where *Trait*_0_ and *Trait* denote the population trait values at 10.00 and 20.00, respectively. Similar to growth rate, functional traits reflect a rate of change over the fluctuation period to assess how traits shift irrespective of their preceding values.

2.1 $$\mathrm{Growth}\;\mathrm{rate}\;(h^{-1})=\frac{\ln(N)-\ln(N_{0})}{t-t_{0}}$$

and

2.2 $$\mathrm{Functional}\;\mathrm{trait}\;(\mathrm{proportional}\;\;\mathrm{change})=\frac{Trait-{Trait}_{0}}{{Trait}_{0}}$$

To standardize responses, all traits were normalized by subtracting the mean stable temperature control (Control) value of each day from individual observed values for each fluctuating regime (R-1114, R-2224, I-1124, I-2114); a positive/negative relative trait value indicates that the trait is higher/lower compared to the mean Control condition.

To determine whether growth rates of the fluctuating regimes had deviated significantly from their respective Control value, independent Wilcoxon rank sum tests were performed to compare the fourth and fifth fluctuations of each regime to its respective Control. This same approach was also used to determine whether growth rates on days without fluctuations (i.e. intervals between fluctuations where temperature returns to 18°C) differed from the Control. These tests were used in place of parametric tests due to the data not meeting assumptions of normality and homogeneity of variance.

To assess *T. pseudonana*'s response during the first phase of regimes, relative trait values during the fourth fluctuation were tested for divergence using the Kruskal–Wallis H test. Pairwise regime comparisons for significant results were performed using independent Wilcoxon rank sum tests with a Bonferroni correction. To assess *T. pseudonana*'s response during phase two, relative trait values during the fifth fluctuation were assessed using this same approach.

To determine if the ‘integrated phenotype’ (combined effect of all traits) had diverged between the regular and irregular regimes, changes in the phenotype were visualized through time using the ordination ‘principal components analysis' (PCA), which was performed separately for each fluctuation on a standardized correlation matrix (mean = 0, s.d. = 1). As the PCA highlighted distinct separation between the regular and irregular regimes, differences between these two groups were verified using tests of homogeneity of dispersions (PERMDISP) and permutational analysis of variance (PERMANOVA), respectively. These analyses test for differences in the geometric spread (PERMDISP) and centre location (PERMANOVA) of these groups, and were performed using a resemblance matrix calculated by Euclidean distance on normalized relative trait data (mean = 0, s.d. = 1) with 999 permutations.

To assess whether *T. pseudonana* hardened in response to recurrent temperature fluctuations, linear regression was used to describe the relative growth trajectories of regimes up to the fourth and fifth fluctuations. Relative trait trajectories were also assessed using this approach.

All data analyses and plotting were performed using R v. 4.0.4 [39] and the following packages: ‘car’ [40], ‘broom’ [41], ‘dplyr’ [42], ‘factoextra’ [43], ‘ggplot2’ [44], ‘ggpubr’ [45], ‘ggtext’ [46], ‘tidyr’ [47], ‘vegan’ [48] and ‘xlsx’ [49]. The datasets associated with this research article are available from the Dryad Digital Repository [50].

## 3. Results

### (a) Growth under fluctuating temperature regimes

**Figure 1.**
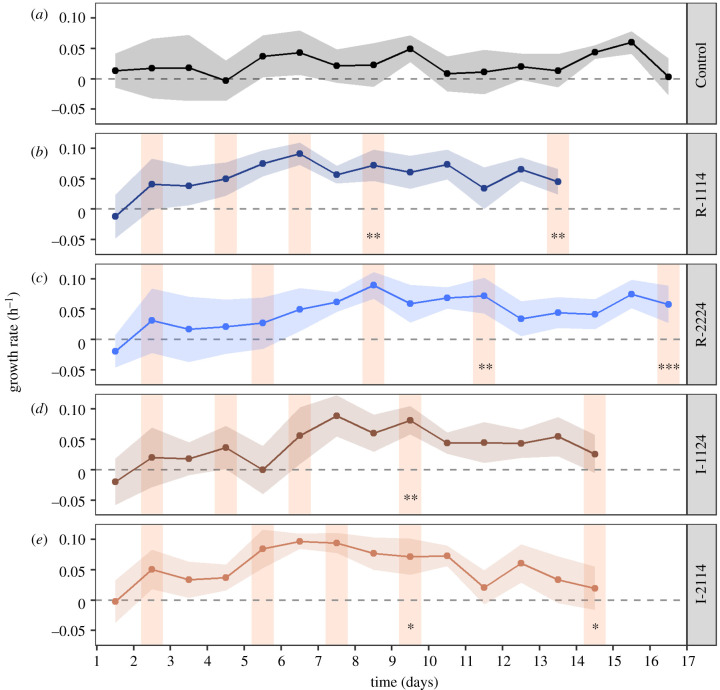
Mean ± s.d. growth rates (h^−1^) of *Thalassiosira pseudonana* across the fluctuation period through time for each of the thermal regimes (*n* = 12 per regime). Orange shaded columns show days corresponding to the 10 h fluctuation exposure to 23°C. Significant comparisons between the Control and the first and second phases of the fluctuating regimes are denoted by the asterisks (independent Wilcoxon rank sum tests, *p < 0.05; **p < 0.01; ***p < 0.001). (Online version in colour.)

Growth of *T. pseudonana* varied in all regimes, even under stable temperature conditions (Control) (figure 1). In the first phase of the experiment (fluctuation 4), growth in the fluctuating regimes was always significantly higher than the Control (figure 1*b–e*). In the second phase (fluctuation 5), growth in the regular regimes was at least twice as high as the Control (figure 1*b,c*), while irregular regimes displayed growth that was similar to or lower than the Control (figure 1*d,e*). Growth on days without fluctuations (when cells returned to 18°C) was generally higher than the Control in all four regimes (independent Wilcoxon rank sum tests: *p* < 0.003 in all cases) (figure 1*a–e*).

### (b) Effect of environmental predictability

**Figure 2.**
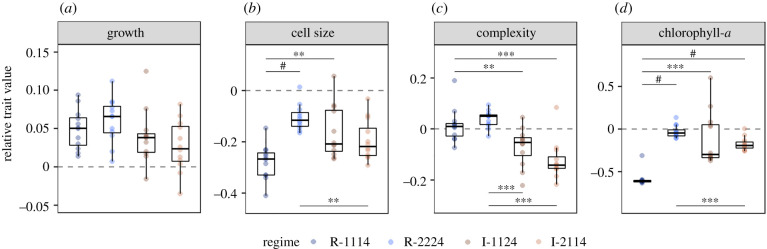
Relative trait values of *Thalassiosira pseudonana* at fluctuation 4 for each of the fluctuating regimes (*n* = 12 per regime) ((*a*) growth, (*b*) cell size, (*c*) complexity and (*d*) chlorophyll-*a*). Points are coloured by regime. Significant comparisons are denoted by the bars and asterisks (independent Wilcoxon rank sum tests with Bonferroni correction, ^#^*p* < 0.0001, ***p < 0.001, **p < 0.01, *p < 0.05). (Online version in colour.)

During the first phase, the growth of *T. pseudonana* populations among the four fluctuating regimes was similar (0.078 ± 0.031 h^−1^) (mean ± s.d.) (Kruskal–Wallis: $\chi_{3}{\;}^{2}=7.09$, *p* = 0.07) (figure 2*a*). This indicates that there was little effect of environmental predictability on instantaneous population growth rates after 8–11 days of regular and irregular sequences of fluctuations. Despite this, there were substantial differences in functional traits (Kruskal–Wallis: *p* < 0.001 in all cases) (figure 2*b–d*).

While cell complexity changed in all regimes by 3–10%, the regularity of fluctuations had a distinct impact. Populations in R-1114 and R-2224 experienced a slight increase in complexity relative to the stable temperature Control (of 2 ± 7 and 4 ± 3%, respectively), whereas those in I-1124 and I-2114 underwent a relative decrease (−7 ± 8 and −12 ± 8%, respectively) (figure 2*c*). Notably, this is the only trait of the four measured that specifically showed an effect of predictability, rather than simply an effect of environmental fluctuation that was observed for cell size and pigmentation (figure 2*b,d*).

### (c) Effect of environmental history

**Figure 3.**
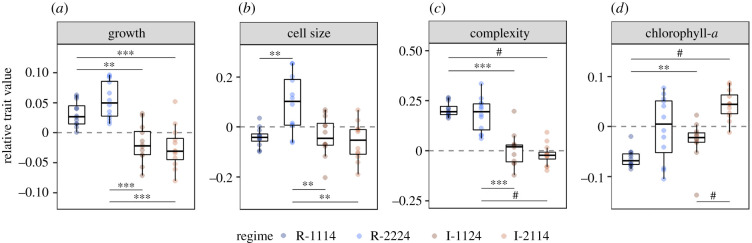
Relative trait values of *Thalassiosira pseudonana* at fluctuation 5 for each of the fluctuating regimes (*n* = 12 per regime) ((*a*) growth, (*b*) cell size, (*c*) complexity and (*d*) chlorophyll-a). Points are coloured by regime. Significant comparisons are denoted by the bars and asterisks (independent Wilcoxon rank sum tests with Bonferroni correction, ^#^*p* < 0.0001, ***p < 0.001, **p < 0.01, *p < 0.05). (Online version in colour.)

During the second phase of the experiment, *T. pseudonana* populations diverged significantly in all traits (Kruskal–Wallis: *p* < 0.01 in all cases), indicating a strong effect of prior thermal history. The trend observed in cellular complexity during the first phase of the experiment (figure 2*c*) continued into the second phase, where the regular and irregular regimes were distinct (figure 3*c*). During this final fluctuation, the complexity of all cells increased by 4–10%; however, cells in I-1124 and I-2114 exhibited little change relative to the Control (a difference of 1 ± 8 and −2 ± 5%, respectively), whereas cells in R-1114 and R-2224 became much more complex (21 ± 4 and 18 ± 9%, respectively).

Notably, we also observed this impact of predictability on growth (figure 3*a*). During this final fluctuation, growth rates ranged between a minimum of −0.036 h^−1^ (mortality) and maximum of 0.099 h^−1^. However, R-1114 and R-2224 grew approximately 3–18 times faster than the Control, with relative rates of 0.031 ± 0.021 and 0.055 ± 0.031 h^−1^ respectively, and cells in I-1124 and I-2114 grew approximately 0.5 times slower than the Control (−0.018 ± 0.032 and −0.024 ± 0.036 h^−1^, respectively). The other traits behaved similarly to phase one, where relative changes in the size and pigmentation of cells differed indiscriminately across regimes (figure 3*b*,*d*).

### (d) The integrated phenotype

**Figure 4.**
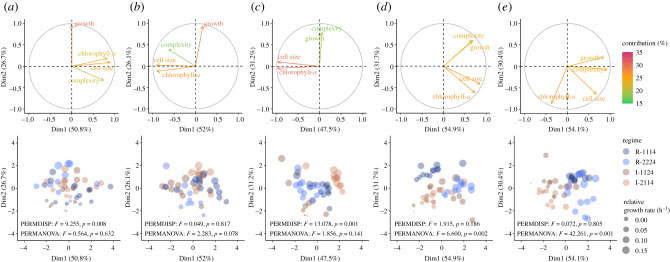
PCA showing the integrated phenotype of *Thalassiosira pseudonana* across fluctuations 1–5 (subplots (*a*–*e*)) among the fluctuating regimes (*n* = 12 per regime). The first row of circular biplots depicts the contributions of each trait to the principal components, where trait vectors pointing in the same direction are highly correlated (and vice versa). The second row of biplots depicts the relative similarity among regimes, where points closer together are more similar phenotypes (and vice versa). Point sizes represent relative growth rates and are coloured by regime. Significant PERMDISP (heterogeneity of dispersions) and PERMANOVA (heterogeneity of centre location) comparisons between the regular and irregular regimes are indicated on each subplot. (Online version in colour.)

Considering all the traits together, there was significant plasticity in *T. pseudonana* phenotypes across regimes. The once homogeneous population (figure 4*a*) became increasingly partitioned, with phenotypes separating into regular and irregular regimes by fluctuation 4 (figure 4*d*). This effect became more pronounced at the fifth fluctuation (figure 4*e*). Dispersion varied in some of the earlier fluctuations indicating phenotypes were more heterogeneous (figure 4*a*,*c*); however, by fluctuation 4 there was a clear distinction between phenotypes of the regular and irregular regimes (figure 4*d*), and by fluctuation 5, the clustering between regimes was most pronounced (figure 4*e*). Overall, the distribution of each phenotype in PCA space remained fairly consistent from fluctuations 1–5, where 81.08 ± 4.12% of variation in phenotypes was accounted for in the first two principal components. This indicates that while the regimes invoke sorting of traits between regular and irregular treatments, the absolute trait variation remained relatively constant.

The relationships among *T. pseudonana* traits shifted as fluctuations ensued. Changes to the size and pigmentation of cells were strongly associated with one another throughout the first four fluctuations (figure 4*a–d*) until they became decoupled during fluctuation 5 (figure 4*e*). Similar trends were observed in the relationships between cell size and complexity, and pigmentation and complexity, though with decoupling during fluctuation 3 (figure 4*a–e*). By contrast, population growth and cellular complexity displayed little association with one another at the first fluctuation (figure 4*a*); however, by the third, they had become strongly correlated (figure 4*c*), a trend that persisted until the final fluctuation (figure 4*e*). The relationships between growth and changes in cell size and pigmentation were generally weak throughout the experiment (figure 4*a–e*).

### (e) Hardening and trait changes under recurrent fluctuations

**Figure 5.**
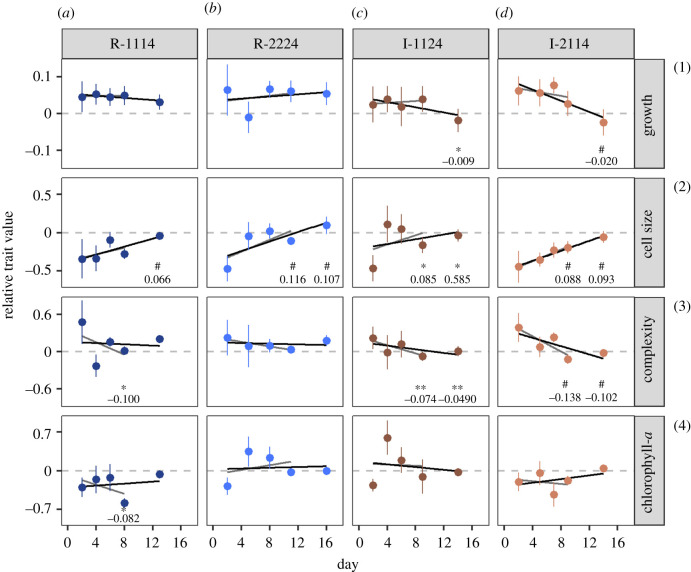
Mean ± s.d. trait values (relative to the Control) of *Thalassiosira pseudonana* in response to fluctuations 1–5 for each of the fluctuating regimes (*n* = 12 per regime). Lines of best fit depict linear regression models over fluctuations 1–4 (phase one, grey line) and 1–5 (phase two, black line), respectively. Stars indicate significance of fitted model (^#^*p* < 0.0001, ***p < 0.001, **p < 0.01, *p < 0.05) and numbers the regression coefficient. Significance indicators are positioned directly below the appropriate phase. (Online version in colour.)

There was no evidence that populations hardened, i.e. had increasingly positive growth during successive fluctuations. Relative growth rates showed no positive change with recurrent fluctuations (figure 5*a–d*1), despite ROS indicating that the +5°C fluctuation was perceived as mildly stressful (electronic supplementary material, figure S3) but with no immediate mortality (figure 1).

There was, however, some evidence of cumulative change with successive fluctuations. Cells in irregular regimes showed a consistent decline in complexity at both phases of the experiment (figure 5*c–d*3) and a decline in growth at phase two (figure 5*c–d*1). Cells in all regimes except R-1114 phase one increased in size (figure 5*a–d*2). However, with the exception of cell size in I-2114, trait values did not show monotonic linear changes.

## 4. Discussion

Our empirical data reveal two main outcomes of temperature fluctuations: (i) the predictability of temperature fluctuations had a distinct impact on phytoplankton growth and traits, and (ii) phenotypic changes and negative growth impacts induced by thermal fluctuations occurred after 13–16 days, corresponding to approximately 23–29 generations (based on a mean generation time of approximately 1.8 d^−1^ in the stable reference environment). This time frame is ecologically relevant given that many marine systems experience monospecific diatom blooms or growing seasons over similar periods [51,52]. Such alteration in growth rate or trait values could influence pertinent oceanic processes such as rates of photosynthetic carbon fixation, seasonal species successions, trophic energy transfer and/or downwards particle transport [28]. Therefore, the outcomes of this study have significant implications for global ocean productivity and atmospheric carbon sequestration, particularly under a warming and increasingly unpredictable climate [1].

Temperature fluctuations generally increased growth rates relative to stable reference environments, even during periods when temperature was not elevated. This was expected given that higher growth rates are observed during fluctuations over accelerating portions of the thermal performance curve [53], with 23°C approaching the 25°C *T*_opt_ for this species [33]. In *T. pseudonana*, thermal fluctuations of this nature may accelerate adaptation to ocean warming under climate change, given that higher growth rates increase the probability of producing mutations beneficial for surviving in warmer environments [54]. However, for taxa with lower *T*_opt_ where temperature increases would expose them to supra-optimal conditions, thermal fluctuations could strongly impact population growth and increase the risk of extinctions. Further research using organisms with different thermal niches, where the impact of fluctuations may be less favourable, is required. Two strategies that diatoms may adopt in response to warming include becoming a warm-adapted specialist, or a thermal generalist [55]. Thus, while the capacity of *T. pseudonana* to adapt to ocean warming by increasing its growth is promising, we also observed a significant interaction with predictability/history. Populations exposed to irregular fluctuations grew at a similar rate or slower than those in stable reference environments during the final fluctuation. By contrast, cells exposed to regular fluctuations showed increased growth. This indicates that predicting responses to future warming is complex and warrants further research to understand the interaction between environmental predictability/history and mean temperature. In particular, experiments large enough to include a gradient of predictabilities that can separate the organismal response to the magnitude of environmental variation from its regularity (as in [15]) are needed to produce generalizable results. This will help facilitate an understanding of how growth rate scales with environmental predictability over the full range of current and anticipated variation in the ocean.

The first sign of divergence in the phenotypes of regular versus irregular regimes was detected within 8–11 days. This shift was linked to adjustments in complexity that may relate to structural or morphological changes of *T. pseudonana* cells, impacting on cellular optical properties. This can be indicative of altered/loss of structures required for protection (via siliceous cell wall ornamentation, shape, outline, thickness, gaps and striation pattern), buoyancy regulation and nutrient storage (via presence and size of internal vacuoles) [56,57]. Thus, a loss of complexity has the potential to impact growth and survival of planktonic diatoms in various ways, including (i) decreased ability to use or avoid patchy nutrient, light and temperature conditions, (ii) diminished intracellular nutrient stores and subsequent ability to reproduce when external nutrients are depleted and (iii) increased susceptibility to predation by lack of physical protection [58]. On the other hand, it is plausible that this decrease in complexity facilitates survival under sub-optimal conditions by limiting cellular activity to only that necessary for immediate survival, thus producing energetically more affordable cells. One way in which phytoplankton may achieve an energy reduction is by entering physiological dormancy through resting stages [59]. Given the uncertainty in what functional changes are linked to adjustments in SSC, both in *T. pseudonana* and phytoplankton more broadly, future work should include additional trait measurements such as cell wall, internal cell and resting stage morphology (e.g. scanning electron/phase contrast/inverted microscopy, lipid and fatty acid quantification [60] etc.), all of which could affect *T. pseudonana*'s ecological and biogeochemical functioning.

When populations are periodically exposed to a stressful environment, two types of responses commonly occur: hardening or stress accumulation. In the timescale assessed in this study (approx. 2 weeks), predictable environmental change had the potential to promote anticipation and greater plasticity in *T. pseudonana* populations as fluctuations proceeded [61,62]. Given that cells in the stable reference environment had a generation time of approximately 1.8 d^−1^, cell division occurred during and/or between fluctuations, indicating that transgenerational effects were at least partially responsible for the differences we observed in the final fluctuation. Under highly predictable scenarios, such plasticity in the parent generation may offer protection to offspring [10]. However, greater unpredictability can lead to transfer/inheritance of maladaptive traits and/or decreased fitness [61], as was observed in this study. While the fluctuations yielded distinct differences in *T. pseudonana*'s phenotype, we found little evidence of heat hardening. Rather, we observed evidence of persistence under regular fluctuations and lagged stress effects under irregular fluctuations. Lagged stress responses clearly indicate carry-over effects which are not immediately detectable following the stress event(s) [25], similar to the delayed response of trees to drought where mortality is often observed some years later [63,64]. Lagged responses to environmental stimuli have also been observed in phytoplankton, such as bloom formation after nutrient input days prior [65]. This lagged stress effect could become an important component of the microbial response to environmental stress in the future, particularly under a more unpredictable climate, with implications for monitoring and predictions of future marine productivity.

Our current understanding of how organisms respond to changes in environmental predictability is based on a small but growing number of empirical studies. There is still, however, a lack of experiments with many key organisms and relevant ranges of patterns and magnitudes of environmental fluctuation for those organisms, particularly in the context of how varying the timing of perturbations influences population demography. While other studies have varied the magnitude, frequency and/or number of fluctuations in environmental variables [5,15,66], to the best of our knowledge, this is one of few to alter temporal *regularity*. In addition, our experimental design was informed by a global ocean simulation and used an organism for which the patterns and magnitude of temperature fluctuation were relevant. Our study shows that for this marine organism, the impacts of differences in thermal predictability occur as soon as 8 days or approximately 15 generations. Not only do microbial populations in the ocean experience divergent thermal histories over a similar time frame, they have also been demonstrated to have significantly different physiological performance [62]. Putting these observations together, this suggests that planktonic organisms experience temperature variation *in-situ* with measurable impacts to population size and phenotype, both of which could influence their evolution [54].

In this study, we found that the predictability of transient warming events affected both cell division rates and other traits in a model diatom. In generalizing from this study, several aspects of the model organism and specific environmental change used need to be taken into account. *T. pseudonana* is a thermal generalist, but many phytoplankton species do not have this breadth of thermal performance [33], such that *T. pseudonana* may be particularly well adapted to growth in fluctuating environments relative to diatom species found primarily in narrower temperature niches. Given that we were able to detect a significant consequence of unpredictability in this thermally robust organism, this raises concern for how other less tolerant species may respond to an ocean that is becoming increasingly unpredictable [1]. The general qualitative response found in our empirical data is still informative, but the magnitude of effects may vary substantially between diatoms, both at the species and intra-species level [33,67]. In particular, thermal specialists will experience more stress for a given increase in temperature, which raises the possibility that lagged stress responses could be both more extreme and more common than indicated in this study.

We use a magnitude of temperature fluctuation that is consistent with global analyses that showed warming of greater than 4°C per day that can be sustained over a 5-day period for plankton arriving in the North Atlantic [29]. Thus, it is not unreasonable to assume that five +5°C fluctuations within a two-week time frame is within the range of variation experienced along global planktonic trajectories. We recommend that future work include both generalists and specialists to understand the mechanisms behind the relationship between changes to environmental predictability and trait values in marine phytoplankton. These experiments should monitor beyond the final fluctuation to assess recovery and determine whether traits converge back to their original states, thereby providing additional insight into the resilience of these organisms under an increasingly unpredictable climate.

## Data Availability

The data supporting this research article are available from the Dryad Digital Repository: https://doi.org/10.5061/dryad.pnvx0k6p2 [50]. Additional methods and figures are provided in the electronic supplementary material [68].
